# Ceftriaxone Relieves Trigeminal Neuropathic Pain Through Suppression of Spatiotemporal Synaptic Plasticity *via* Restoration of Glutamate Transporter 1 in the Medullary Dorsal Horn

**DOI:** 10.3389/fncel.2020.00199

**Published:** 2020-06-30

**Authors:** Xiao Luo, Ting He, Yan Wang, Jiang-Lin Wang, Xue-Bin Yan, Hao-Cheng Zhou, Rui-Rui Wang, Rui Du, Xiao-Liang Wang, Jun Chen, Dong Huang

**Affiliations:** ^1^Department of Pain Management, The Third Xiangya Hospital, Institute of Pain Medicine, Central South University, Changsha, China; ^2^Institute for Biomedical Sciences of Pain, Tangdu Hospital, The Fourth Military Medical University, Xi’an, China; ^3^Key Laboratory of Brain Stress and Behavior, People’s Liberation Army, Xi’an, China; ^4^Department of Pain Management, The Affiliated Hospital of Southwest Medical University, Luzhou, China

**Keywords:** trigeminal neuropathic pain, glutamate transporter 1, ceftriaxone, synaptic plasticity, long-term potentiation, medullary dorsal horn

## Abstract

Using a rat model of trigeminal neuropathic pain (TNP) produced by chronic compression of the infraorbital nerve (CCI-ION), we investigated the analgesic effect and the underlying mechanisms of ceftriaxone (Cef), a β-lactam antibiotic, that is thought to be a potent stimulator of glutamate transporter 1 (GLT-1). First, repeated intraperitoneal (i.p.) injections of Cef (200 mg/kg) for 5-days since Day 1 of CCI-ION could significantly relieve both mechanical and thermal pain hypersensitivity from day 10 after drug administration. Western blot and immunofluorescent results demonstrated that 5-days administration of Cef resulted in the restoration of GLT-1 expression to a level equivalent to the sham control which was dramatically lost under the TNP condition. Moreover, multi-electrode (8 × 8) array recordings of network field excitatory postsynaptic potentials (fEPSPs) were performed on the acutely dissociated medullary dorsal horn slice evoked by electrical stimulation of the trigeminal spinal tract. The results showed that the increased number of fEPSPs, induction rate, and maintenance of long-term potentiation caused by CCI-ION were significantly suppressed by 5-days administration of Cef. Taken together, the results indicate that Cef can relieve TNP through suppression of spatiotemporal synaptic plasticity *via* GLT-1 restoration in the medullary dorsal horn of the trigeminal nerve.

## Introduction

Trigeminal neuralgia (TN) is one of the most intense orofacial pain affecting one or more branches of the 5th cranial nerve (Finnerup et al., 2010; Scholz et al., 2019). The causes and pathology of trigeminal neuralgia are not well understood and the treatment is still a major challenge. Carbamazepine is the first-line drug recommended by the American Academy of Neurology and the European Federation of Neurological Societies which can effectively alleviate TN, however, the side-effects become apparent with the emergence of drug resistance (Finnerup et al., 2010; Scholz et al., 2019). A substantial proportion of patients have to abandon the drug and choose surgical treatment. The treatment will become extremely troublesome for the patients with recurrent symptoms after surgery, while the recurrent rate ranges from 20% to 50% 5 years after surgery (Tronnier et al., 2001; Cheng et al., 2014). So, more effective and novel drugs are required to replace the traditional ones.

Glutamate transporter 1 (GLT-1) is a sodium-dependent high-affinity glutamate transporter which is abundantly localized in astrocytes and axon-terminals in some parts of the mature brain (Rothstein et al., 1994; Chen et al., 2002, 2004; Furness et al., 2008; Rose et al., 2009). In the spinal cord, GLT-1 is also largely found in astrocytes and growing axonal fibers especially in the gray matter (Rothstein et al., 1994; Danbolt, 2001; Yan et al., 2014). Glutamate is the major excitatory neurotransmitter participating in nociceptive processing and synaptic plasticity during which trafficking of ionotropic glutamate receptors may occur (Malinow and Malenka, 2002; Larsson and Broman, 2011; Liu and Salter, 2010). Both release and uptake abnormalities of glutamate were observed in neuropathic pain (Inquimbert et al., 2012; Di Cesare Mannelli et al., 2015). GLT-1 is crucial for the modulation of the dynamic balance of neurotransmitters in the synaptic cleft, together with glutamate/aspartate transporter (GLAST) to accomplish more than 95% of glutamate uptaking (Danbolt, 2001; Kanai et al., 2013). In case of GLT-1 down-regulation, the extracellular glutamate concentrations would be increased, resulting in excitotoxic neuronal damage that can be happening in the process of amyotrophic lateral sclerosis (Rothstein et al., 1995), spinal trauma (Rao et al., 1998; Lepore et al., 2011) and neuropathy (Jacob et al., 2007; Hazell et al., 2010).

Previous studies have demonstrated that the abnormal expression of GLT-1 was associated with the development of neuropathic pain in various animal models, such as chronic constriction injury of the sciatic nerve (SN-CCI; Hu et al., 2010; Suzuki et al., 2012), ligation of spinal nerves (SNL; Hobo et al., 2011) and spared nerve injury (SNI) of the sciatic nerve (Inquimbert et al., 2012). A transient up-regulation of GLT-1 was observed in some pain models at the initial phase (Yamada et al., 1998; Cavaliere et al., 2007), which was thought to be a compensatory effect in response to the increasing glutamate release. However, the expression of GLT-1 was stably down-regulated at the later phase of the peripheral nerve injury (Weng et al., 2014; Yan et al., 2014). The down-regulation of GLT-1 contributes to an increase in glutamate concentration at the synaptic cleft, which may result in the activation of distant spinal neurons and astrocytic cells (Nie and Weng, 2010; Nie et al., 2010). Excessive extracellular glutamate accumulation has been shown to trigger intracellular Ca^2+^ release in astrocytic cells (Rojas et al., 2007; Yoshizumi et al., 2012), that may magnify the primary afferents. Based on the findings above, GLT-1 is regarded as a promising therapeutic target in neuropathic pain (Hu et al., 2010; Ramos et al., 2010; Nicholson et al., 2014; Chelini et al., 2017; Butler, 2018).

Growing evidence indicates that astrocytes regulate synaptic transmission and plasticity through modulation of excitatory-inhibitory balance, especially the increased efficacy of glutamatergic neurotransmission (Bonansco and Fuenzalida, 2016). Under the neuropathic conditions, such as epilepsy, glutamate-mediated gliotransmission was considered to be a putative signal of increased neuronal excitability (Tang and Lee, 2001). By uptaking glutamate in the synaptic cleft, glutamate transporter is likely to maintain excitatory-inhibitory balance and prevent aberrant signals which may trigger a cascade of plasticity events. The previous studies have proved that GLT-1 plays a critical role in LTP induction and maintenance of long-term changes of synaptic efficacy (Katagiri et al., 2001; Levenson et al., 2002). However, the precise regulation of GLT-1 in astrocyte-neuron communication and synaptic plasticity remains to be explored. It is thus hypothesized that nerve injury-induced GLT-1 changes may alter the spatiotemporal characteristics and coding of nociceptive signals.

The β-lactam antibiotic ceftriaxone (Cef), which can increase the expression of GLT-1 *in vivo* and *in vitro* (Rothstein et al., 2005). In previous studies, Cef was shown to reverse the down-regulation of GLT-1 and to elevate the glutamate uptake in chronic pain models, subsequently resulting in an analgesic effect (Hu et al., 2010; Nicholson et al., 2014; Butler, 2018). The compounds of the Cef are expected to be a class of promising analgesic drugs that can be in combination with opioid analgesics to reduce tolerance (Rawls et al., 2010). Pharmacological inhibition of GLT-1 by dihydrokainate can reverse the analgesic effect of Cef in diabetic rats (Gunduz et al., 2011). Moreover, Cef can exert its therapeutic effect by reducing glial activation in neuropathic pain (Ramos et al., 2010; Nicholson et al., 2014). However, the exact therapeutic effects of Cef and its modulation of the glutamate transporter system in the TN is still not clear. Therefore, we designed experiments and looked at the following aspects of Cef: (1) anti-nociceptive effects of long-term administration of Cef in a rat model of TN; (2) effects of long-term administration of Cef on the expression level of GLT-1 in the trigeminal nucleus of the spinal tract caudalis (Sp5C), which is known as the medullary dorsal horn receiving primary nociceptive afferents from the semilunar ganglion sensory cells of the trigeminal nerve; (3) effects of long-term administration of Cef on the spatiotemporal network responses of excitatory postsynaptic field potentials (fEPSPs) evoked by electrical stimulation of the Sp5 recorded on medullary dorsal horn slice using multi-electrode array (MEA, 8 × 8) recording system.

## Materials and Methods

### Animals and Surgery

Experiments were carried out on male albino Sprague–Dawley rats (3–4 weeks old, 80–120 g) provided by the Laboratory Animal Center of Fourth Military Medical University (FMMU). Animals were housed in a group of five per cage under standard laboratory conditions (12:12 h day/night cycle, with a temperature of 22–26°C and air humidity of 55–60%), with access to food and water *ad libitum*. All the experiments were approved by the Institutional Animal Care and Use Committee at the FMMU and were as per the recommendations of the ARRIVE guidelines, the U.K. Animals (Scientific Procedures) Act 1986 and associated guidelines, the EU Directive 2010/63/EU for animal experiments, the National Institutes of Health Guide for Care and Use of Laboratory Animals (Publication No. 85–23, revised 1996). The ethical guidelines for investigations of experimental pain in conscious animals of the International Association for the Study of Pain were also critically followed. Efforts have been made to minimize the number of animals used and their suffering.

**Figure 1:**
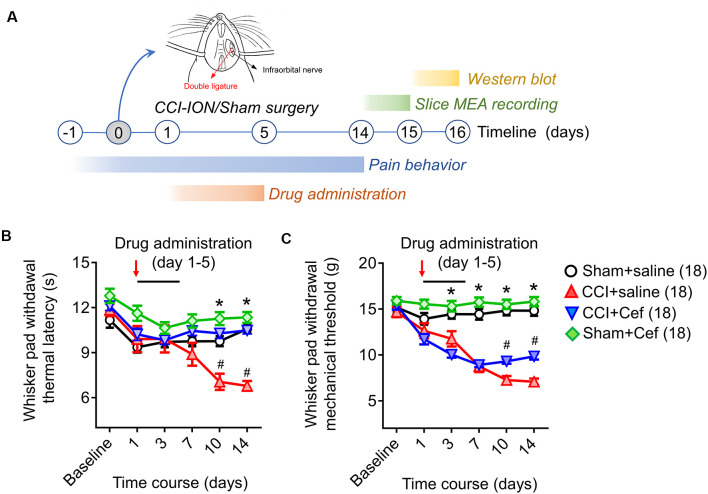
Timeline of the experimental design and procedures, as well as the effects of repeated systemic administration of ceftriaxone (Cef) on CCI-ION-induced pain hypersensitivity. **(A)** Timeline of the experimental design and procedures (for details see “Materials and Methods” section in the text). **(B,C)** Changes in whisker pad withdrawal thermal latency and mechanical threshold in rats with Sham + saline (*n* = 18), CCI-saline (*n* = 18), CCI-Cef (*n* = 18) and Sham + Cef (*n* = 18). Arrows indicate the start of the drug administration. Data are presented as mean ± SEM. *p < 0.05, CCI + saline vs. Sham + saline; ^#^*p* < 0.05, CCI + Cef vs. CCI + saline. CCI-ION, chronic constriction injury of infraorbital nerve; MEA, multi-electrode array.

To prepare a model of trigeminal neuropathic pain (TNP), unilateral ligation of the infraorbital nerve (ION) was performed following the protocols described by Imamura (Imamura et al., 1997). The rats were anesthetized with pentobarbital sodium (50 mg/kg, i.p.) and an incision was made intraorally along the gingivobuccal margin to expose the ION (Figure 1A). A double ligature was tied loosely around the ION with 5–0 chromic guts (2 mm apart). After ligation, the incision was sutured using silk. In sham-operated rats, the ION was isolated using the same procedure except that the nerve was not ligated.

### Behavioral Tests

The behavioral assays were performed before and post-surgery from day 1 to day 14 (Figure 1A). The rats were individually placed in a plastic cage 1 h before the test to adapt to the environment. A set of von Frey filaments were used to measure the mechanical sensitivity of the facial whisker pad as previously described, according to the up-and-down method with the cut-off intensity of 15 g (Vos et al., 1994). The thermal sensitivity of the facial whisker pad was evaluated using the published method (Imamura et al., 1997). A special box with a hole at the front which allows the rat’s snout to poke through was prepared, besides the hole, the other part of the front-end was covered with a white paper that occluded the rat’s vision when its snout protruded through the hole. The thermal stimulus was given to the centra of the whisker pad that can raise the skin temperature to 45–50°C in 10 s. The cut-off time was set at 20 s to prevent tissue damage. Thermal withdrawal latency was measured three times for each rat at intervals of 2 min.

### Slice Preparation

Rats were anesthetized with pentobarbital sodium (50 mg/kg, i.p.) and then perfused with oxygenated N-methyl-D-glucamine (NMDG) artificial cerebrospinal fluid (NMDG ACSF) containing 92 mM NMDG, 2.5 mM KCl, 1.25 mM NaH_2_PO_4_, 30 mM NaHCO_3_, 20 mM HEPES, 25 mM glucose, 2 mM thiourea, 5 mM Na-ascorbate, 3 mM Na-pyruvate, 0.5 mM CaCl_2_, and 10 mM MgCl_2_·6H_2_O (pH 7.3–7.4) according to Ting’s protocol (Ting et al., 2014), the medullary and upper cervical spinal tissue was quickly isolated and submerged into pre-oxygenated NMDG ACSF. The tissue was fixed in an agar stage with cyanoacrylate glue to stick on the plate and then cut into 400 μm coronal slices with a Vibratome (Dosaka, DTK-1000, Japan). Slices were transferred to a chamber continuously perfused with oxygenated HEPES holding ACSF containing 92 mM NaCl, 2.5 mM KCl, 1.25 mM NaH_2_PO_4_, 30 mM NaHCO_3_, 20 mM HEPES, 25 mM glucose, 2 mM thiourea, 5 mM Na-ascorbate, 3 mM Na-pyruvate, 2 mM CaCl_2_, and 2 mM MgCl_2_·6H_2_O. Before MEA recording, slices were recovered for at least 2 h at room temperature.

### Electrophysiological Recordings

The electrophysiological recordings were performed on days 14–15 after surgery (Figure 1A). A 64 (8 × 8) array multi-electrode dish system (MED-64, Alpha-Med Scientific, Japan) was used to record fEPSPs on a medullary slice containing the Sp5C (Figure 4A). After recovery in the HEPES holding ACSF for 2 h, the slice was transferred to the MED-64 probe (P515A, 50 × 50 μm, 150 μm interval) to cover the medullary dorsal horn and a nylon mesh anchor was covered to the slice to ensure stability. The slice was continuously perfused with oxygenated recording ACSF containing 119 mM NaCl, 2.5 mM KCl, 1.25 mM NaH_2_PO_4_, 24 mM NaHCO_3_, 12.5 mM glucose, 2 mM CaCl_2_, and 2 mM MgCl_2_·6H_2_O at a flow rate of 2 ml/min with a peristaltic pump (PERI-STARTM, PI, USA) at room temperature. After 10–15 min adaption, at least one channel was arranged over the Sp5 allowing electrical stimulation with the aid of visual localization through a charge-coupled device camera (DP70, Olympus, Japan). A biphasic square-wave pulse (0.1 Hz, 0.2 ms, 10–199 μA) generated by the data acquisition software (Conductor 3.0, Panasonic Alpha-Med Sciences, Japan) was applied to the electrical stimulus site, the evoked fEPSPs were amplified by the 64-channel amplifier and digitized at 20 kHz sampling rate. The input-output (I-O) curve was acquired by measurements of the fEPSPs amplitude and slope in response to the gradient stimulation from 20 to 199 μA with a 20 μA stepwise increase. Higher intensities (>199 μA) were not applied due to the limitation of the stimulus generator. The stimulus intensity for the test was then adjusted to enable produce 40–60% of maximal response based on the I-O curve. As for long-term potentiation (LTP) induction, the high-frequency stimulation (HFS, 10 bursts/s, 4 × 0.2 ms pulses with 60 μA at 100 Hz for each burst) protocol was used as described before (Heusler et al., 2000; Hjornevik et al., 2008; Zhao et al., 2009). The test stimulus was repeatedly delivered once every 10 min for 90 min and the responses were measured as an average of five individual traces. In another set of experiments, CNQX (10 μM) and (TTX 0.5 μM) were perfused to the slices at a rate of 2 ml/min to identify whether the network field potentials are ionic glutamate receptor-mediated fEPSPs. After that, the slices were washed by fresh ACSF and tested until the responses tended to be stable. The details of the MED-64 system usage can be referred to our previous publications (Zhao et al., 2009; Wang et al., 2010; Yu et al., 2017).

**Figure 2:**
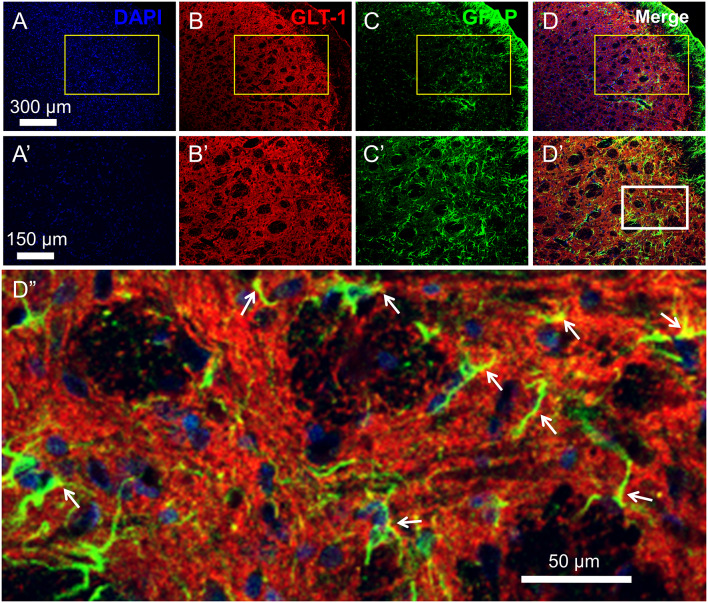
Distribution and co-localization of glutamate transporter 1 (GLT-1) and glia fibrillary acidic protein (GFAP)-labeled profiles in the medullary dorsal horn. Panels **(A–D)** show triple labeling of DAPI (blue), GLT-1 (red) and GFAP (green). Panesl **(A’–D’)** show insets from panels **(A–D)**. Panel **(D”)** shows inset from panel **(D’)**. Scale = 300 μm for **(A–D)**; 150 μm for **(A’–D’)** and 50 μm for **(D”)**. Arrows indicate co-localization of GLT-1 and GFAP (yellow) in astrocytic profiles.

**Figure 3:**
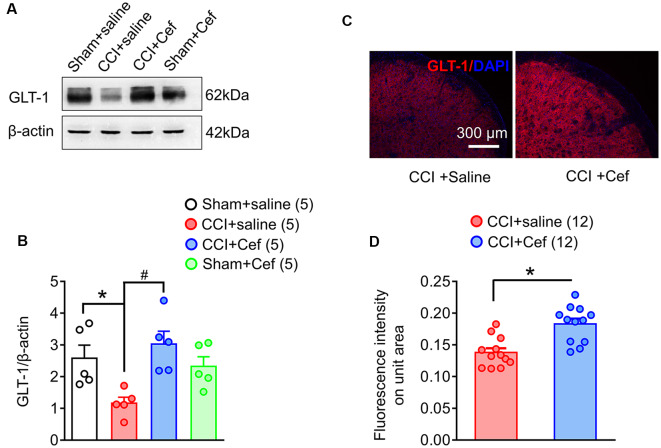
Reversal effects of repeated systemic administration of ceftriaxone (Cef) on CCI-ION-induced loss of GLT-1 protein in the medullary dorsal horn. Panels **(A,B)** show expression of GLT-1 protein in the ipsilateral medullary dorsal horn of rats with Sham + saline (*n* = 5), CCI-saline (*n* = 5), CCI-Cef (*n* = 5) and Sham + Cef (*n* = 5). Data are presented as mean ± SEM. *p < 0.05, CCI + saline vs. Sham + saline; ^#^*p* < 0.05, CCI + Cef vs. CCI + saline. **(C,D)** Immunofluorescent staining of GLT-1 (red) and DAPI (blue) in the ipsilateral medullary dorsal horn of CCI + saline (*n* = 12) and CCI + Cef (*n* = 12) groups. Data are shown as mean ± SEM. *p < 0.05, CCI + Cef vs. CCI + saline. GLT-1, glutamate transporter 1.

**Figure 4:**
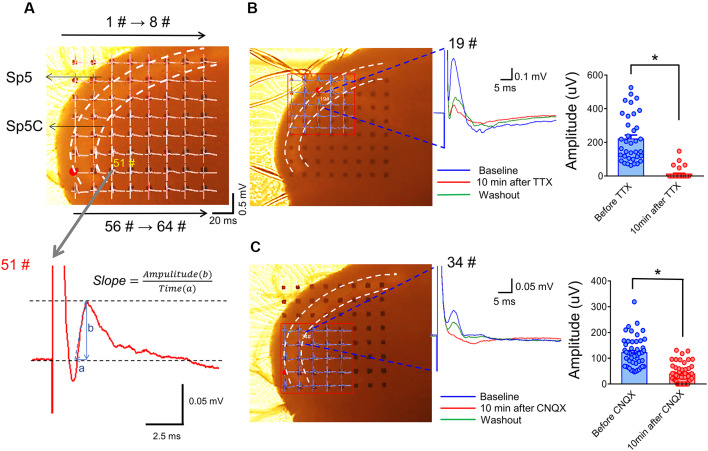
Arrangement of an acutely dissociated medullary dorsal horn slice on an MEA recording dish and pharmacological identification of evoked network local field potentials (eLFPs). Panel **(A)** shows a typical example of the MEA (8 × 8, 64 electrodes) recordings on a rat medullary dorsal horn (Sp5C) slice. The upper panel shows representative network traces of eLFPs recorded across the 63 sites following electrical stimulation at the spinal tract of the trigeminal nerve (Sp5, red dot for #49). The vertical scale indicates the amplitude of potentials (0.5 mV), horizontal scale indicates the time (20 ms). The lower panel showing a typical waveform of eLFPs recorded from 51# electrode (gray arrow), the slope was calculated as the ratio of amplitude (b) to a time interval (a). Panel **(B)** shows blocking effect of eLFPs by perfusion with TTX (1 μM; dots mean 36 electrode points from *n* = 6 slices). Panel **(C)** shows the suppressive effect of eLFPs by perfusion with CNQX (10 μM; dots mean 66 electrode points from *n* = 8 slices). *p < 0.05, drug vs. baseline. Error bars: ±SEM.

For the quantification of the I-O relationship, the amplitude and slope of fEPSPs were analyzed by the MED-64 conductor. The total number of effective fEPSPs (>20% baseline) was counted by an experimenter unaware of the experimental design and averaged across slices for each group. For induction of LTP, a 20% increase in the amplitudes of fEPSPs over the baseline that could be maintained for more than 30 min was set as successful induction (Figure 6B). The amplitudes of fEPSP were normalized and expressed as a percentage to the baseline value.

**Figure 5:**
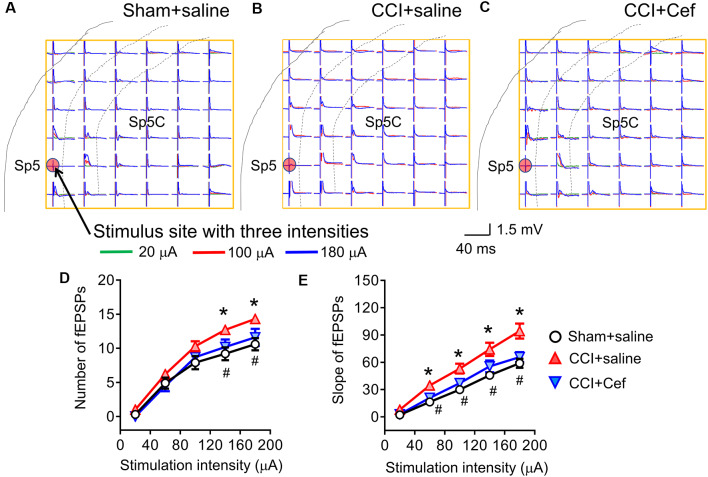
Reversal effects of repeated systemic administration of ceftriaxone (Cef) on CCI-ION-induced enhancement of network field excitatory postsynaptic potentials (fEPSPs) response. Panels **(A–C)** shows typical examples of the MEA (8 × 8, 64 electrodes) recordings on a rat medullary dorsal horn (Sp5C) slice from Sham + saline, CCI + saline and CCI + Cef groups following three intensities (overlapping traces: green for 20 μA, red for 100 μA and blue for 180 μA) of electrical stimulation at the Sp5 (red dot). Panel **(D)** shows the average number of evoked fEPSPs following electrical stimulation with a series of intensities ranging from 20 μA to 199 μA in Sham + saline (*n* = 10), CCI + saline (*n* = 10) and CCI-Cef (*n* = 9) groups. Panel **(E)** shows averaged slope of evoked fEPSPs following electrical stimulation with a series of intensities ranging from 20 μA to 199 μA in Sham + saline (*n* = 10), CCI + saline (*n* = 10) and CCI-Cef (*n* = 9) groups. The data of panels **(D,E)** are presented as mean ± SEM. *p < 0.05, CCI + saline vs. Sham + saline; ^#^*p < 0.05*, CCI + Cef vs. CCI + saline.

**Figure 6:**
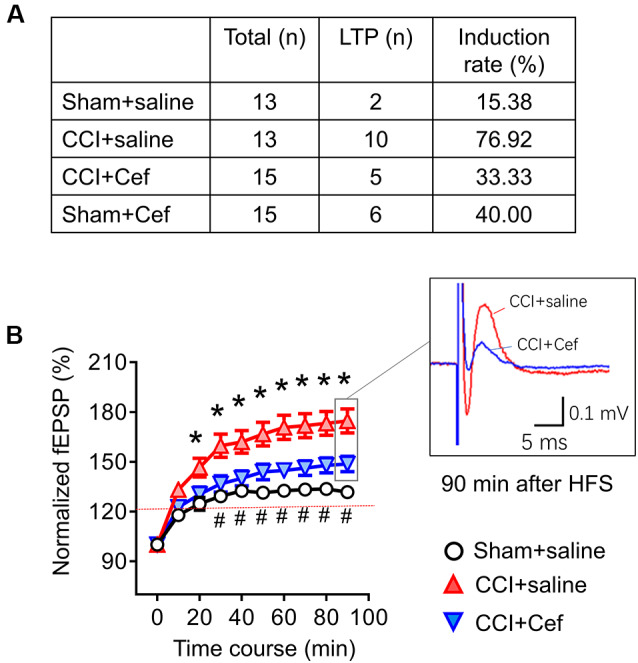
Reversal effects of repeated systemic administration of ceftriaxone (Cef) on CCI-ION-induced enhancement of long-term potentiation (LTP) induction and maintenance. Panel **(A)** shows induction rate of LTP induced by high frequency burst stimulation (10 bursts/s, 4 × 0.2 ms pulses at 100 Hz, 60 μA for each burst) in rats of Sham + saline (*n* = 13), CCI + saline (*n* = 13), CCI-Cef (*n* = 15) and Sham + Cef (*n* = 15) groups. **(B)** Time courses of LTP in rats of Sham + saline (15 electrode points from two rats), CCI-saline (49 electrode points from seven slices), and CCI + Cef (24 electrode points from five slices) groups. Data are presented as mean ± SEM. *p < 0.05, CCI + saline vs. Sham + saline; ^#^*p* < 0.05, CCI + Cef vs. CCI+saline.

### Western Blot

Medullary slices of each group were quickly removed and washed by PBS after MED-64 recording (Figure 1A), and then the tissue was homogenized in RIPA lysis buffer for 10 min in an ice bath. The lysates were centrifuged for 10 min at 12,000 rpm at 4°C. A BCA protein assay kit was used to measure the protein concentration. The samples (50 μg protein) were placed on the SDS-PAGE gel, run at 80 V for 30 min and then 120 V for 1 h, and then transferred to nitrocellulose membrane (Bio-Rad). After block with 5% nonfat milk in PBS for 1 h, the membrane was incubated overnight at 4°C with primary rabbit anti-GLT-1 antibody (1:1,000, Abcam, UK) and mouse anti-β-actin (1:2,000, Abcam). After three times of washing (10 min each) in PBST, the membranes were incubated with secondary HRP-conjugated goat-anti-rabbit antibody (1:2,000, Bio-Rad) for 2 h at room temperature. The membranes were treated with enhanced chemiluminescence solution (Alpha Innotech Corp., USA) and the signals were detected by FluorChem FC2 (Alpha Innotech Corp). The density of each band was measured using a computer-assisted imaging analysis system (Bio-Rad, CA, USA) and normalized to β-actin intensity.

### Immunofluorescence

Tissues were obtained 10 days after CCI-ION surgery. After anesthetized with pentobarbital sodium (50 mg/kg, i.p.), the rats were transcardially perfused with PBS, followed by 4% paraformaldehyde in 0.1 M PB solution. The medulla oblongata was removed and placed in 4% paraformaldehyde overnight at 4°C, and then transferred to 30% sucrose in PBS for cryoprotection. The tissues were cut into coronal sections (25 μm) on CM1900 freezing microtome (Leica, Germany). The sections were rinsed in PBS and blocked with 10% goat serum in PBS for 2 h, and then incubated in primary antibodies overnight at 4°C. Washed by PBS for three times (10 min each), the sections were incubated with secondary antibodies for 2 h in a dark environment at room temperature. The primary antibodies used in the present study were rabbit anti-GLT-1 (1:200, Abcam, UK) and mouse anti-glia fibrillary acidic protein (GFAP; 1:200, Sigma–Aldrich, St. Louis, MO, USA). The secondary antibodies were Cy3 (552 nm) anti-rabbit (1:100, Sigma–Aldrich, St. Louis, MO, USA) and fluorescein isothiocyanate isomer (FITC; 494 nm) anti-mouse. After that, DAPI (1:2,000, Sigma–Aldrich, St. Louis, MO, USA) was added to the sections for 10 min. The sections were washed by PBS and mounted on slides. A laser scan confocal fluorescent microscope (Olympus FV1000, Japan) was used to acquire the images and the fluorescence intensity was measured by an image processing program ImageJ (National Institutes of Health, Bethesda, MD, USA).

### Drugs

The β-lactam antibiotic ceftriaxone sodium (Cef) was purchased from Roche Corp (Switzerland) and dissolved in saline solution (0.9% NaCl) at 0.2 g/mL. The Cef solution was administered by intraperitoneal (i.p.) injection (200 mg/kg). Cef administration started from day 1 after CCI-ION surgery and continued for five consecutive days (Figure 1A).

### Statistics

Data were presented as mean ± SEM. One way ANOVA with LSD *post hoc* corrections and independent-sample *t*-test (two-tailed) was used for parametric data, while Kruskal–Wallis one way ANOVA test with Bonferroni *post hoc* corrections and Mann–Whitney *U*-test were used for non-parametric data according to the results of normality Shapiro-Wilk test and equal variance Levene test, and Pearson Chi-square tests with Fisher exact tests were used for fourfold table data (for details see Supplementary Table S1). All statistical analyses were performed used SPSS 25.0 software. *P* < 0.05 was considered to be statistically significant. The artwork was performed by GraphPad Prism 6.01 (GraphPad Software Inc., San Diego, CA, USA).

## Results

### Anti-nociceptive Effects of Repeated Systemic Administration of Cef on the CCI-ION-Induced Hyperalgesia

Compared with the sham group, rats with CCI-ION developed both thermal and mechanical pain hypersensitivity since 10 days after surgery (Figures 1B,C, *p < 0.001 and *p < 0.001, CCI + saline vs. Sham + saline, for details of statistical analysis see Supplementary Table S1). However, the CCI-ION-induced thermal and mechanical pain hypersensitivity were both suppressed by 5-days i.p. administrations of Cef relative to vehicle control (Figures 1B,C, ^#^*p* < 0.001 and ^#^*p* = 0.001 in day 10, CCI + Cef vs. CCI + saline, for details of statistical analysis see Supplementary Table S1). The same treatment of Cef did not have any effects on the basal mechanical and thermal sensitivity (Figures 1B,C, see Supplementary Table S1). Time course observations revealed that the thermal pain hypersensitivity was more sensitive to the Cef treatment than mechanical pain hypersensitivity (Figures 1B,C).

### Reversal Effects of Repeated Systemic Administration of Cef on CCI-ION-Induced Loss of GLT-1 Expression in the Spinal Dorsal Horn

As previously reported, double immunofluorescent labeling showed co-localization of GLT-1 and GFAP, a specific marker of astrocytes, in the medullary dorsal horn of rats (Figure 2). Compared with sham control, CCI-ION resulted in significant loss of GLT-1 protein expression in the medullary dorsal horn when assayed on day 15 after surgery (Figures 3A,B, *p = 0.009, CCI + saline vs. Sham + saline, see Supplementary Table S1). However, the CCI-ION-induced decrease in GLT-1 expression was fully reversed by 5-days i.p. administrations of Cef relative to vehicle control (Figures 3A–D, *p < 0.001 for immunofluorescent labeling and ^#^*p* = 0.009 for Western blot, CCI + Cef vs. CCI + saline, for details of statistical analysis see Supplementary Table S1). Similar to the behavioral test, the same treatment of the Cef did not have any effects on the level of GLT-1 expression in the sham group of rats (Figures 3A,B, see Supplementary Table S1).

### Reversal Effects of Repeated Systemic Administration of Cef on CCI-ION-Induced Enhanced Network fEPSPs Response and LTP Induction and Maintenance

Figure 4A showed an arrangement of MED-64 recording dish with 64 channels (8 × 8 array) over the medullary dorsal horn slice wherein one channel (see red dot) was remained for electrical stimulation of the Sp5, while the other channels were used for simultaneous recordings of network fEPSPs in the medullary dorsal horn (see lower panel of Figure 4A for the typical waveform of an fEPSP recorded from 51# channel, arrows for 1#–8# and 56#–64# on the upper panel indicate the order of electrode array). The Sp5-activated local network field potentials could be completely blocked by TTX (Figure 4B) and partially blocked by CNQX (Figure 4C), suggesting mediation of network fEPSPs by both action potentials produced by activation of voltage-gated sodium channels (VGSCs) and ionic non-NMDA and probably NMDA glutamate receptors (Zhao et al., 2009; Wang et al., 2010; Yu et al., 2017).

Following 14–15 days after the CCI-ION, the spatial number of the Sp5-evoked fEPSPs was significantly increased when tested by a higher intensity of electrical stimulation (140–200 μA) relative to sham control (Figures 5A,B,D, *p = 0.029 CCI + saline vs. Sham + saline, see Supplementary Table S1). The I-V curve was leftward shifted in a group of CCI-ION relative to the sham control as well (Figures 5A,B,E, *p = 0.004 CCI + saline vs. Sham + saline, see Supplementary Table S1). However, repeated i.p. treatment with Cef reversed both the CCI-ION-expanded spatial network fEPSPs response (Figures 5B–D, ^#^*p* = 0.027 CCI + Cef vs. CCI + saline, see Supplementary Table S1) and CCI-ION-produced leftward-shift of I-V curve (Figures 5B,C,E, ^#^*p* = 0.027 CCI + Cef vs. CCI + saline, see Supplementary Table S1), compared to the vehicle control.

As for the effects of Cef-treatment on the induction and maintenance of LTP, conditioning stimulation was applied through electrical stimulation electrode (10 bursts/s, 4 × 0.2 ms pulses at 100 Hz, 60 μA for each burst). As shown in Figure 6A, the induction rate of LTP was also dramatically increased in the CCI-ION group by about 77% relative to 15.4% in the sham group (*p* = 0.005, CCI + saline vs. Sham + Saline, see Supplementary Table S1). However, the increased induction rate of LTP was distinctly reversed by repeated systemic treatment of Cef (Figure 6A, *P* = 0.030, CCI + Cef vs. CCI + Saline: 33.33% vs. 76.92%, see Supplementary Table S1). Cef-treatment could not change the induction rate in sham control (Figure 6A, *P* = 0.221, Sham + Cef vs. Sham + Saline: 40.00% vs. 15.38%, see Supplementary Table S1). The CCI-ION resulted in enhancement of LTP in terms of both amplitude and time course relative to the sham control (Figure 6B, *P* = 0.001, CCI + Saline vs. Sham + Saline, see Supplementary Table S1), while repeated systemic treatment of Cef produced a reversal effect on such enhancement compared to the vehicle control (Figure 6B, *P* = 0.024, CCI + Cef vs. CCI + Saline, see Supplementary Table S1).

## Discussion

The major gains of the current study are as follows: (1) repeated systemic administration of Cef could mitigate both orofacial thermal and mechanical pain hypersensitivity caused by CCI-ION in rats, while the same treatment did not have any effects on the basal pain sensitivity, suggesting a potential therapeutic use of the drug in prevention of clinical-pathological pain; (2) the therapeutic effect of Cef on the CCI-ION-induced TN was through restoration of GLT-1 expression in the medullary dorsal horn that had been deficient during the neuropathic processes; (3) the GLT-1 deficiency could lead to dysfunctional synaptic plasticity in the medullary dorsal horn, displaying as enhancement of both spatial widespread and temporal elongation of network synaptic responses in terms of increased number of fEPSPs, leftward shift of I-V curve, and enhanced LTP induction and maintenance; and (4) repeated systemic administration of Cef could reverse the abnormal spatiotemporal synaptic plasticity through restoration of GLT-1 expression in the medullary dorsal horn under the CCI-ION-induced trigeminal neuropathic condition.

### Contribution of Medullary Dorsal Horn GLT-1 Deficiency to the Development of Trigeminal Neuropathic Pain

Glutamate serves as a major excitatory neurotransmitter in the central nervous system (CNS) and plays a critical role in excitatory synaptic transmission *via* acting at its ionic (AMPA/NMDA) and/or metabotropic receptors (Ozawa et al., 1998). It is well known that both peripheral tissue and nerve injuries can cause central sensitization or abnormal synaptic plasticity in the spinal dorsal horn that involves glutamatergic neurotransmission (Woolf and Thompson, 1991; Larsson and Broman, 2011). However, over the past decades, a huge number of studies have been focused on the therapeutic strategy targeting antagonists and blockers of various subtypes of glutamate receptors but with fewer being concentrating on how to restore dysfunctions of glutamate release, reuptake and metabolism during the processes of pathological pain. Long-term abnormal extracellular elevation of spinal glutamate release has been previously found to be caused by peripheral inflammatory pain (Yan et al., 2009). The imbalance between excitatory and inhibitory synaptic transmission and modulation has also been revealed to be existent in various CNS regions due to increased excitatory amino acids (EAAs) and decreased inhibitory amino acids (IAAs) under both neuropathic and inflammatory pain conditions (Yan et al., 2009; Gong et al., 2010; Wang et al., 2018; Cao et al., 2019). Those previous results highly suggest that abnormal reuptake of presynaptic glutamate release by astrocytic GLT-1 or GLAST may lead to synaptic cleft accumulation of EAAs that persistently activate post-synaptic AMPA/NMDA receptors and downstream intracellular cascades, resulting in enhancement of EPSPs associated with chronic pain (Danbolt, 2001; Kanai et al., 2013). As lines of evidence, astrocytic GLT-1 expression could be impaired by both spinal cord and peripheral nerve injuries, leading to down-regulation of GLT-1 protein and an increase in extracellular glutamate accumulation (Rao et al., 1998; Jacob et al., 2007; Wang et al., 2018). Moreover, abnormal down-regulation of GLT-1 has also been demonstrated to be associated with the development of neuropathic pain induced by CCI-SN (Suzuki et al., 2012), SNL (Hobo et al., 2011) and SNI (Inquimbert et al., 2012). Elsewhere, a transient up-regulation of GLT-1 was observed in some pain models at the initial phase (Yamada et al., 1998; Cavaliere et al., 2007), which was thought to be a compensatory effect in response to the increasing glutamate release, however, the expression of GLT-1 was stably down-regulated at the later phase of the peripheral nerve injury (Weng et al., 2014; Yan et al., 2014), implicating potential contribution of medullary dorsal horn GLT-1 deficiency to development of TNP. In the current study, we provided a new line of evidence supporting this presumption by different levels of assays in terms of behavioral, cellular and molecular, and electrophysiological techniques. Meanwhile, we demonstrated that repeated systemic administration of Cef for at least 5 days could restore protein expression of GLT-1 at the medullary dorsal horn wherein the CCI-ION-induced abnormal spatiotemporal synaptic plasticity was subsequently rescued and pain hypersensitivity was greatly improved. Because the rescue effects of Cef to up-regulate GLT-1 expression and to increase glutamate reuptake have also been demonstrated to be responsible for the relief of neuropathic pain due to other origins (Hu et al., 2010; Ramos et al., 2010; Butler, 2018) and repeated systemic administration of Cef did not affect basal pain sensitivity in normal and sham rats, this class of structural analogs might be promising in the management of neuropathic pain in the clinic. Although repeated administration of Cef at the acute phase (day 1 after the CCI-ION) was demonstrated to be effective in the prevention of the TNP condition from occurring in the current study, it is still unknown whether the same Cef treatment would be effective to relieve chronic phase of the TNP. Thus, a future study is required to be carried out to look at the therapeutic analgesic effect of Cef under the chronic phase of the TNP.

### Contribution of Medullary Dorsal Horn GLT-1 Deficiency to Abnormal Spatiotemporal Synaptic Plasticity Induced by Pathological Pain

It is well known that both tissue and nerve injury in the periphery can cause spatiotemporal synaptic plasticity in the CNS of different levels from spinal dorsal horn to the primary somatosensory cortex (S1), anterior cingulate cortex (ACC) and hippocampus (Gong et al., 2010; Lyu et al., 2013; Lu et al., 2014; Yu et al., 2017; Wang et al., 2018; Cao et al., 2019). The spatiotemporal synaptic plasticity when abnormally lasts can be associated with development and maintenance of chronic pain and its comorbidities including emotional disorders (anxiety and depression) and cognitive deficits (Gong et al., 2010; Lyu et al., 2013; Liu and Chen, 2014; Lu et al., 2014; Yu et al., 2017; Wang et al., 2018; Cao et al., 2019). At the single synaptic level, slice whole-cell recordings have demonstrated that the frequency of miniature and spontaneous excitatory postsynaptic currents (mEPSCs/sEPSCs) could be greatly increased, while that of inhibitory postsynaptic currents (mIPSCs/sIPSCs) could be distinctly decreased under pathological pain condition, implicating an imbalance between EAAs and IAAs release from pre-synaptic component (Gong et al., 2010; Wang et al., 2018; Cao et al., 2019). This imbalance between EAAs and IAAs releases at the synaptic cleft is likely to be responsible for abnormal spatiotemporal synaptic plasticity displaying as the enhanced amplitude of fEPSPs, the leftward shift of I-V curve and increased LTP induction rate and magnitude (Zhao et al., 2009; Gong et al., 2010; Lyu et al., 2013; Lu et al., 2014; Wang et al., 2018; Cao et al., 2019). Moreover, the pathological pain associated imbalance between EAAs and IAAs releases at the synaptic cleft was able to cause translocation or trafficking of GluR1 from cytosol to cell membrane (i.e., externalization), while GluR2 and GABA_A_1α from the membrane to the cytosol (i.e., internalization), facilitating induction and magnification of LTP in association with NMDA receptors (Cao et al., 2019). In the current study, we further demonstrated that the CCI-ION could produce spatiotemporal synaptic plasticity at the medullary dorsal horn as well displaying an enhanced amplitude of fEPSPs, the leftward shift of I-V curve and increased LTP induction rate and magnitude. The CCI-ION-induced spatiotemporal synaptic plasticity could be fully reversed by repeated systemic administration of Cef which could restore the GLT-1 expression, suggesting a contribution of medullary dorsal horn GLT-1 deficiency to abnormal spatiotemporal synaptic plasticity induced by neuropathic pain. Namely, the CCI-ION-induced medullary dorsal horn GLT-1 deficiency would lead to the accumulation of glutamate at the synaptic cleft, resulting in persistent activation of ionic AMPA/NMDA receptors and intracellular cascades that are required for LTP induction and maintenance. This process may be exaggerated by magnified primary afferents (Sp5) maintained by excessive extracellular glutamate accumulation-triggered intracellular Ca^2+^ release in astrocytic cells (Rojas et al., 2007; Yoshizumi et al., 2012). It is well known that the rate of diffusion is one of the major parameters in glutamate clearance (Piet et al., 2004), and this process is accelerated by glutamate transporters (e.g., GLT-1) expressed in astrocytic cells (Tzingounis and Wadiche, 2007). The abnormal deficiency of glutamate transporters could directly influence the clearance of glutamate, subsequently leading to glutamate spills over the synaptic cleft and diffuses to distant synapses. At the spatial network level, because previous studies have shown that widespread activation of distant neurons and astrocytic cells could be elicited by widespread extracellular glutamate accumulation due to the GLT-1 deficiency in the spinal dorsal horn (Nie and Weng, 2010; Nie et al., 2010), network widespread expansion of fEPSPs observed in our current and previous studies (Zhao et al., 2009; Lyu et al., 2013; Lu et al., 2014; Cao et al., 2019) could be well explained. Of course, activation of glutamate mGluRs, the release of GABA_A_-mediated tonic inhibition and activation of silent synapses could also be involved in this processing (Voronin and Cherubini, 2004).

As for the reversal effect of Cef on the CCI-ION-induced GLT-1 deficiency, little is known about its mechanistic actions. In the current study, it was revealed that repeated systemic administration of Cef could up-regulate the expression of GLT-1 in the medullary dorsal horn, suggesting its direct or indirect genetic or epigenetic modulation of GLT-1 genes in certain astrocytic cells. Some previous studies have found the phenomenon that Cef could relieve neuropathic pain through up-regulation of GLT-1, but it could not fully explain how Cef works at the molecular and cellular levels by some presumptions, i.e., astrocytic inactivation (Nicholson et al., 2014) or astrocytic-neuronal bidirectional communications (Pasti et al., 1997; Ni et al., 2007). Theoretically, direct or indirect genetic or epigenetic modulation of GLT-1 genes in certain astrocytic cells by Cef could be explainable for the 10-days delayed analgesic effects following 5-days systemic administration of Cef observed in the current study. The underlying mechanisms of Cef-induced restoration of GLT-1 expression remain unclear and require to be further studied in the future.

In summary: Cef can relieve TNP through suppression of spatiotemporal synaptic plasticity *via* GLT-1 restoration in the medullary dorsal horn of the trigeminal nerve and is promising as a novel therapeutic target for the treatment of TN, in particular, and other neuropathic pains, in general.

## Data Availability Statement

All datasets presented in this study are included in the article/Supplementary Material.

## Ethics Statement

The animal study was reviewed and approved by Institutional Animal Care and Use Committee at the Fouth Military Medical University.

## Author Contributions

DH and JC designed and managed the research work. XL, TH, J-LW, R-RW, and X-LW performed the experiments and collected the data. XL, TH, YW, X-BY, H-CZ, RD, and JC contributed to the analyses and plotting of the data. JC, XL, and DH composed the manuscript.

## Supplementary Material

The Supplementary Material for this article can be found online at: https://www.frontiersin.org/articles/10.3389/fncel.2020.00199/full#supplementary-material.

## Conflict of Interest

The authors declare that the research was conducted in the absence of any commercial or financial relationships that could be construed as a potential conflict of interest.
