# Supplementary material for: Ceftriaxone Relieves Trigeminal Neuropathic Pain Through Suppression of Spatiotemporal Synaptic Plasticity via Restoration of Glutamate Transporter 1 in the Medullary Dorsal Horn
Source: Front Cell Neurosci. 2020 Jun 30;14:199. doi: 10.3389/fncel.2020.00199 (PMC7340123; doi:10.3389/fncel.2020.00199)
Supplement: Supplementary file 1 [file Table_1.DOC]

Table S1 Number of samples, statistical tests and *p* values for Figs.1-4

| **Figure number** | **Number of animals or samples** | **Normality test** | **Equal variance test** | **Statistical method** | ***p* value** | ***F*/*t* value** | **P value with post hoc correction** |
| --- | --- | --- | --- | --- | --- | --- | --- |
| Fig.1B | | | | | | | |
| 1B PWTL_baseline | n = 18 for sham+saline  n = 18 for CCI+saline  n = 18 for CCI+CEF  n = 18 for sham+CEF | failed |  | K-W one way ANOVA test | *p* = 0.231 | *H_C_* = 4.296 |  |
|  |  |  |  | Mann-Whitney *U* test (two-tailed) post hoc comparision |  |  | sham+saline vs CCI+saline:*p* = 0.327;  CCI+saline vs CCI+CEF: *p* = 0.486;  sham+saline vs sham+CEF: *p* = 0.088 |
| 1B PWTL_day1 | n = 18 for sham+saline  n = 18 for CCI+saline  n = 18 for CCI+CEF  n = 18 for sham+CEF | passed | failed | Welch’s ANOVA test | *p* = 0.062 | *F*= 2.662 |  |
|  |  |  |  | Dunnett T3 post hoc comparison |  |  | sham+saline vs CCI+saline:*p* = 1.000;  CCI+saline vs CCI+CEF: *p* = 1.000;  sham+saline vs sham+CEF: *p* = 0.061 |
| 1B PWTL_day3 | n = 18 for sham+saline  n = 18 for CCI+saline  n = 18 for CCI+CEF  n = 18 for sham+CEF | passed | failed | Welch’s ANOVA test | *p* = 0.312 | *F*= 1.233 |  |
|  |  |  |  | Dunnett T3 post hoc comparison |  |  | sham+saline vs CCI+saline:*p* = 1.000;  CCI+saline vs CCI+CEF: *p* = 1.000;  sham+saline vs sham+CEF: *p* = 0.398 |
| 1B PWTL_day7 | n = 18 for sham+saline  n = 18 for CCI+saline  n = 18 for CCI+CEF  n = 18 for sham+CEF | passed | failed | Welch’s ANOVA test | *p* = 0.037 | *F*= 3.138 |  |
|  |  |  |  | Dunnett T3 post hoc comparison |  |  | sham+saline vs CCI+saline:*p* = 0.876;  CCI+saline vs CCI+CEF: *p* = 0.338;  sham+saline vs sham+CEF: *p* = 0.103 |
| 1B PWTL_day10 | n = 18 for sham+saline  n = 18 for CCI+saline  n = 18 for CCI+CEF  n = 18 for sham+CEF | passed | passed | One-way ANOVA test | *p* < 0.001 | *F*_0.05, (3,68)_ = 20.450 |  |
|  |  |  |  | Bonferroni post hoc comparison |  |  | sham+saline vs CCI+saline:*p* < 0.001;  CCI+saline vs CCI+CEF: *p* < 0.001;  sham+saline vs sham+CEF: *p* = 0.012 |
| 1B PWTL_day14 | n = 18 for sham+saline  n = 18 for CCI+saline  n = 18 for CCI+CEF  n = 18 for sham+CEF | passed | passed | One-way ANOVA test | *p* < 0.001 | *F*_0.05, (3,68)_ = 44.092 |  |
|  |  |  |  | Bonferroni post hoc comparison |  |  | sham+saline vs CCI+saline:*p* < 0.001;  CCI+saline vs CCI+CEF: *p* < 0.001;  sham+saline vs sham+CEF: *p* = 0.483 |
| Fig.1C | | | | | | | |
| 1C PWMT_baseline | n = 18 for sham+saline  n = 18 for CCI+saline  n = 18 for CCI+CEF  n = 18 for sham+CEF | failed |  | K-W one way ANOVA test | *p* = 0.360 | *H_C_* = 3.216 |  |
|  |  |  |  | Mann-Whitney *U* test (two-tailed) post hoc comparision |  |  | sham+saline vs CCI+saline:*p* = 0.497;  CCI+saline vs CCI+CEF: *p* = 0.341;  sham+saline vs sham+CEF: *p* = 0.257 |
| 1C PWMT_day1 | n = 18 for sham+saline  n = 18 for CCI+saline  n = 18 for CCI+CEF  n = 18 for sham+CEF | failed |  | K-W one way ANOVA test | *p* < 0.001 | *H_C_* = 18.619 |  |
|  |  |  |  | Mann-Whitney *U* test (two-tailed) post hoc comparision |  |  | sham+saline vs CCI+saline:*p* = 0.245;  CCI+saline vs CCI+CEF: *p* = 0.279;  sham+saline vs sham+CEF: *p* = 0.065 |
| 1C PWMT_day3 | n = 18 for sham+saline  n = 18 for CCI+saline  n = 18 for CCI+CEF  n = 18 for sham+CEF | failed |  | K-W one way ANOVA test | *p* < 0.001 | *H_C_* = 32.656 |  |
|  |  |  |  | Mann-Whitney *U* test (two-tailed) post hoc comparision |  |  | sham+saline vs CCI+saline:*p* = 0.014;  CCI+saline vs CCI+CEF: *p* = 0.060;  sham+saline vs sham+CEF: *p* = 0.123 |
| 1C PWMT_day7 | n = 18 for sham+saline  n = 18 for CCI+saline  n = 18 for CCI+CEF  n = 18 for sham+CEF | failed |  | K-W one way ANOVA test | *p* < 0.001 | *H_C_* = 49.305 |  |
|  |  |  |  | Mann-Whitney *U* test (two-tailed) post hoc comparision |  |  | sham+saline vs CCI+saline:*p* < 0.001;  CCI+saline vs CCI+CEF: *p* = 0.714;  sham+saline vs sham+CEF: *p* = 0.050 |
| 1C PWMT_day10 | n = 18 for sham+saline  n = 18 for CCI+saline  n = 18 for CCI+CEF  n = 18 for sham+CEF | failed |  | K-W one way ANOVA test | *p* < 0.001 | *H_C_* = 55.811 |  |
|  |  |  |  | Mann-Whitney *U* test (two-tailed) post hoc comparision |  |  | sham+saline vs CCI+saline:*p* < 0.001;  CCI+saline vs CCI+CEF: *p* = 0.001;  sham+saline vs sham+CEF: *p* = 0.135 |
| 1C PWMT_day14 | n = 18 for sham+saline  n = 18 for CCI+saline  n = 18 for CCI+CEF  n = 18 for sham+CEF | failed |  | K-W one way ANOVA test | *p* < 0.001 | *H_C_* = 55.260 |  |
|  |  |  |  | Mann-Whitney *U* test (two-tailed) post hoc comparision |  |  | sham+saline vs CCI+saline:*p* < 0.001;  CCI+saline vs CCI+CEF: *p* < 0.001;  sham+saline vs sham+CEF: *p* = 0.157 |
| Fig.3B | | | | | | | |
| Fig.3B GLT-1/β-actin protein | n = 5 for sham+saline  n = 5 for CCI+saline  n = 5 for CCI+CEF  n = 5 for sham+CEF | failed |  | K-W one way ANOVA test | *p* = 0.008 | *H_C_* = 9.780 |  |
|  |  |  |  | Mann-Whitney *U* test(two-tailed) |  |  | sham+saline vs CCI+saline:*p* = 0.009;  CCI+saline vs CCI+CEF: *p* = 0.009;  sham+saline vs sham+CEF: *p* = 0.602 |
| Fig.3D | | | | | | | |
| Fig.3D GLT-1 Fluorescence intensity | n = 12 for CCI+saline （12 slices from 3 rats ）  n = 12 for CCI+CEF （12 slices from 3 rats ） | passed | passed | Independent sample T test (two-tailed) | *p* < 0.001 | *t =* -4.256 |  |
| Fig.4 | | | | | | | |
| Fig.4B TTX amplitude | n = 36 for before TTX （36 eletrodes from 6 rats ）  n = 36 for after TTX  （36 eletrodes from 6 rats ） | failed |  | Wilcoxon  Signed Rank  Test (twotailed) | *p* < 0.001 | *Z=* -4.636 |  |
| Fig.4C CNQX amplitude | n = 66 for before CNQX  （66 eletrodes from 8 rats ）  n = 66 for after CNQX  （66 eletrodes from 8 rats ） | failed |  | Wilcoxon  Signed Rank  Test (twotailed) | *p* < 0.001 | *Z=* -9.763 |  |
| Fig.5D | | | | | | | |
| 5D Number of fEPSP _20 uA | n = 10 for sham+saline  n = 10 for CCI+saline  n = 9 for CCI+CEF | failed |  | K-W one way ANOVA test | *p* = 0.100 | *H_C_* = 4.615 |  |
|  |  |  |  | Mann-Whitney *U* test (two-tailed) post hoc comparision |  |  | sham+saline vs CCI+saline:*p* = 0.396;  CCI+saline vs CCI+CEF: *p* = 0.039; |
| 5D Number of fEPSP _60 uA | n = 10 for sham+saline  n = 10 for CCI+saline  n = 9 for CCI+CEF | failed |  | K-W one way ANOVA test | *p* = 0.321 | *H_C_* = 2.273 |  |
|  |  |  |  | Mann-Whitney *U* test (two-tailed) post hoc comparision |  |  | sham+saline vs CCI+saline:*p* = 0.236;  CCI+saline vs CCI+CEF: *p* = 0.170; |
| 5D Number of fEPSP _100 uA | n = 10 for sham+saline  n = 10 for CCI+saline  n = 9 for CCI+CEF | passed | passed | One-way ANOVA test | *p* = 0.177 | *F*_0.05, (2,26)_ = 1.851 |  |
|  |  |  |  | Bonferroni post hoc comparison |  |  | sham+saline vs CCI+saline:*p* = 0.220;  CCI+saline vs CCI+CEF: *p* = 0.595; |
| 5D Number of fEPSP _140 uA | n = 10 for sham+saline  n = 10 for CCI+saline  n = 9 for CCI+CEF | passed | passed | One-way ANOVA test | *p* = 0.025 | *F*_0.05, (2,26)_ = 4.266 |  |
|  |  |  |  | Bonferroni post hoc comparison |  |  | sham+saline vs CCI+saline:*p* = 0.029;  CCI+saline vs CCI+CEF: *p* = 0.137; |
| 5D Number of fEPSP _180 uA | n = 10 for sham+saline  n = 10 for CCI+saline  n = 9 for CCI+CEF | failed |  | K-W one way ANOVA test | *p* = 0.018 | *H_C_* = 8.052 |  |
|  |  |  |  | Mann-Whitney U test (two-tailed) post hoc comparision |  |  | sham+saline vs CCI+saline:*p* = 0.008;  CCI+saline vs CCI+CEF: *p* = 0.027; |
| Fig.5E | | | | | | | |
| 5E Slope of fEPSP _20 uA | n = 10 for sham+saline  n = 10 for CCI+saline  n = 9 for CCI+CEF | failed |  | K-W one way ANOVA test | *p* < 0.001 | *H_C_* = 18.798 |  |
|  |  |  |  | Mann-Whitney *U* test (two-tailed) post hoc comparision |  |  | sham+saline vs CCI+saline:*p* = 0.001;  CCI+saline vs CCI+CEF: *p* < 0.001; |
| 5E Slope of fEPSP _60 uA | n = 10 for sham+saline  n = 10 for CCI+saline  n = 9 for CCI+CEF | failed |  | K-W one way ANOVA test | *p* = 0.009 | *H_C_* = 9.440 |  |
|  |  |  |  | Mann-Whitney *U* test (two-tailed) post hoc comparision |  |  | sham+saline vs CCI+saline:*p* = 0.003;  CCI+saline vs CCI+CEF: *p* = 0.027; |
| 5E Slope of fEPSP _100 uA | n = 10 for sham+saline  n = 10 for CCI+saline  n = 9 for CCI+CEF | failed |  | K-W one way ANOVA test | *p* = 0.010 | *H_C_* = 9.272 |  |
|  |  |  |  | Mann-Whitney *U* test (two-tailed) post hoc comparision |  |  | sham+saline vs CCI+saline:*p* = 0.004;  CCI+saline vs CCI+CEF: *p* = 0.027; |
| 5E Slope of fEPSP _140 uA | n = 10 for sham+saline  n = 10 for CCI+saline  n = 9 for CCI+CEF | failed |  | K-W one way ANOVA test | *p* = 0.066 | *H_C_* = 5.422 |  |
|  |  |  |  | Mann-Whitney *U* test (two-tailed) post hoc comparision |  |  | sham+saline vs CCI+saline:*p* = 0.013;  CCI+saline vs CCI+CEF: *p* = 0.253; |
| 5E Slope of fEPSP _180 uA | n = 10 for sham+saline  n = 10 for CCI+saline  n = 9 for CCI+CEF | passed | failed | Welch’s ANOVA test | *p* = 0.013 | *F*= 5.715 |  |
|  |  |  |  | Dunnett T3 post hoc comparison |  |  | sham+saline vs CCI+saline:*p* = 0.013;  CCI+saline vs CCI+CEF: *p* = 0.029; |
| Fig.6A | | | | | | | |
| 6A LTP induced rate | n = 13 for sham+saline  n = 13 for CCI+saline  n = 15 for CCI+CEF  n = 15 for sham+CEF |  |  | Pearson Chi-square tests, with Fisher exact test when T<5. | *p* < 0.001 | *χ^2^=*127.795 | sham+saline vs CCI+saline:*p* = 0.005, *χ^2^=*9.905;  CCI+saline vs CCI+CEF: *p* = 0.030, *χ^2^=*5.320;  sham+saline vs sham+CEF: *p* = 0.221 *χ^2^=*2.068. |
| Fig.6B |  |  |  |  |  |  |  |
| 6B Normalized fEPSP of LTP_10min | n = 15 for sham+saline  （15 eletrodes from 2 rats）  n = 49 for CCI+saline  （49 eletrodes from 7 rats ）  n = 24 for CCI+CEF  （24 eletrodes from 5 rats ） | failed |  | K-W one way ANOVA test | *p=* 0.085 | *HC =*4.921 |  |
|  |  |  |  | Mann-Whitney U test (two-tailed) post hoc comparision |  |  | sham+saline vs CCI+saline:*p* = 0.054;  CCI+saline vs CCI+CEF: *p* = 0.103; |
| 6B Normalized fEPSP of LTP_20min | n = 15 for sham+saline  （15 eletrodes from 2 rats）  n = 49 for CCI+saline  （49 eletrodes from 7 rats ）  n = 24 for CCI+CEF  （24 eletrodes from 5 rats ） | failed |  | K-W one way ANOVA test | *p=* 0.053 | *HC =* 5.865 |  |
|  |  |  |  | Mann-Whitney U test (two-tailed) post hoc comparision |  |  | sham+saline vs CCI+saline:*p* = 0.036;  CCI+saline vs CCI+CEF: *p* = 0.083; |
| 6B Normalized fEPSP of LTP_30min | n = 15 for sham+saline  （15 eletrodes from 2 rats）  n = 49 for CCI+saline  （49 eletrodes from 7 rats ）  n = 24 for CCI+CEF  （24 eletrodes from 5 rats ） | failed |  | K-W one way ANOVA test | *p=* 0.009 | *HC =* 9.377 |  |
|  |  |  |  | Mann-Whitney U test (two-tailed) post hoc comparision |  |  | sham+saline vs CCI+saline:*p* = 0.009;  CCI+saline vs CCI+CEF: *p* = 0.024; |
| 6B Normalized fEPSP of LTP_40min | n = 15 for sham+saline  （15 eletrodes from 2 rats）  n = 49 for CCI+saline  （49 eletrodes from 7 rats ）  n = 24 for CCI+CEF  （24 eletrodes from 5 rats ） | failed |  | K-W one way ANOVA test | *p=* 0.009 | *HC =* 9.442 |  |
|  |  |  |  | Mann-Whitney U test (two-tailed) post hoc comparision |  |  | sham+saline vs CCI+saline:*p* = 0.005;  CCI+saline vs CCI+CEF: *p* = 0.040; |
| 6B Normalized fEPSP of LTP_50min | n = 15 for sham+saline  （15 eletrodes from 2 rats）  n = 49 for CCI+saline  （49 eletrodes from 7 rats ）  n = 24 for CCI+CEF  （24 eletrodes from 5 rats ） | failed |  | K-W one way ANOVA test | *p =* 0.002 | *HC =* 12.850 |  |
|  |  |  |  | Mann-Whitney U test (two-tailed) post hoc comparision |  |  | sham+saline vs CCI+saline:*p* = 0.001;  CCI+saline vs CCI+CEF: *p* = 0.033; |
| 6B Normalized fEPSP of LTP_60min | n = 15 for sham+saline  （15 eletrodes from 2 rats）  n = 49 for CCI+saline  （49 eletrodes from 7 rats ）  n = 24 for CCI+CEF  （24 eletrodes from 5 rats ） | failed |  | K-W one way ANOVA test | *p =* 0.001 | *HC =* 14.222 |  |
|  |  |  |  | Mann-Whitney U test (two-tailed) post hoc comparision |  |  | sham+saline vs CCI+saline:*p* = 0.001;  CCI+saline vs CCI+CEF: *p* = 0.024; |
| 6B Normalized fEPSP of LTP_70min | n = 15 for sham+saline  （15 eletrodes from 2 rats）  n = 49 for CCI+saline  （49 eletrodes from 7 rats ）  n = 24 for CCI+CEF  （24 eletrodes from 5 rats ） | failed |  | K-W one way ANOVA test | *p* < 0.001 | *HC =* 16.457 |  |
|  |  |  |  | Mann-Whitney U test (two-tailed) post hoc comparision |  |  | sham+saline vs CCI+saline: *p* <0.001;  CCI+saline vs CCI+CEF: *p* = 0.017; |
| 6B Normalized fEPSP of LTP_80min | n = 15 for sham+saline  （15 eletrodes from 2 rats）  n = 49 for CCI+saline  （49 eletrodes from 7 rats ）  n = 24 for CCI+CEF  （24 eletrodes from 5 rats ） | failed |  | K-W one way ANOVA test | *p* < 0.001 | *HC =* 16.900 |  |
|  |  |  |  | Mann-Whitney U test (two-tailed) post hoc comparision |  |  | sham+saline vs CCI+saline: *p* <0.001;  CCI+saline vs CCI+CEF: *p* = 0.019; |
| 6B Normalized fEPSP of LTP_90min | n = 15 for sham+saline  （15 eletrodes from 2 rats）  n = 49 for CCI+saline  （49 eletrodes from 7 rats ）  n = 24 for CCI+CEF  （24 eletrodes from 5 rats ） | failed |  | K-W one way ANOVA test | *p* < 0.001 | *HC =* 22.318 |  |
|  |  |  |  | Mann-Whitney U test (two-tailed) post hoc comparision |  |  | sham+saline vs CCI+saline: *p* <0.001;  CCI+saline vs CCI+CEF: *p* = 0.009; |

Notes: PWTL, (whisker) pad withdrawal thermal latencies; PWMT, (whisker) pad withdrawal mechanical thresholds; CCI, chronic compression injury;Cef, ceftriaxone; K-W, Kruskal-Wallis; GLT-1, glutamate transporter 1 ; TTX, tetrodotoxin; CNQX: 6-Cyano-7-nitroquinoxaline-2,3-dione; fEPSP, field excitatory postsynaptic potentials; LTP, long-term potentiation.
